# Inter-Hospital Transfer after Return of Spontaneous Circulation Shows no Correlation with Neurological Outcomes in Cardiac Arrest Patients Undergoing Targeted Temperature Management in Cardiac Arrest Centers

**DOI:** 10.3390/jcm9061979

**Published:** 2020-06-24

**Authors:** Yoon Hee Choi, Dong Hoon Lee, Je Hyeok Oh, Jin Hong Min, Tae Chang Jang, Won Young Kim, Won Jung Jeong, Je Sung You

**Affiliations:** 1 Department of Emergency Medicine, School of Medicine, Ewha Womans University, Seoul 07804, Korea; unii@ewha.ac.kr; 2 Department of Emergency Medicine, College of Medicine, Chung-Ang University, Seoul 06974, Korea; jehyeokoh@cau.ac.kr; 3 Department of Emergency Medicine, College of Medicine, Chungnam National University, Daejeon 35015, Korea; laphir@cnu.ac.kr; 4 Department of Emergency Medicine, School of Medicine, Daegu Catholic University, Daegu 42472, Korea; emzzang@cu.ac.kr; 5 Department of Emergency Medicine, Ulsan University College of Medicine, Asan Medical Center, Seoul 05505, Korea; wonpia73@naver.com; 6 Department of Emergency Medicine, St. Vincent’s Hospital, College of Medicine, The Catholic University of Korea, Seoul 06591, Korea; medpooh@hanmail.net; 7 Department of Emergency Medicine, Yonsei University College of Medicine, Seoul 03722, Korea; youjsmd@yhs.ac

**Keywords:** inter-hospital transfer, cardiac arrest, neurological outcome, targeted temperature management, post-cardiac arrest syndrome, prognostic factor

## Abstract

This study evaluated whether inter-hospital transfer (IHT) after the return of spontaneous circulation (ROSC) was associated with poor neurological outcomes after 6 months in post-cardiac-arrest patients treated with targeted temperature management (TTM). We used data from the Korean Hypothermia Network prospective registry from November 2015 to December 2018. These out-of-hospital cardiac arrest (OHCA) patients had either received post-cardiac arrest syndrome (PCAS) care at the same hospital or had been transferred from another hospital after ROSC. The primary endpoint was the neurological outcome 6 months after cardiac arrest. Subgroup analyses were performed to determine differences in the time from ROSC to TTM induction according to the electrocardiography results after ROSC. We enrolled 1326 patients. There were no significant differences in neurological outcomes between the direct visit and IHT groups. In patients without ST elevation, the mean time to TTM was significantly shorter in the direct visit group than in the IHT group. IHT after achieving ROSC was not associated with neurologic outcomes after 6 months in post-OHCA patients treated with TTM, even though TTM induction was delayed in transferred patients.

## 1. Introduction

Comprehensive post-resuscitation care is important for survivors of out-of-hospital cardiac arrest (OHCA) [1]. Because the care for post-cardiac arrest syndrome (PCAS) is specialized and complex, various treatment strategies, including a range of specialists and resources, should be available in a multi-disciplinary fashion. When the index hospital to which the victim is initially transported cannot provide comprehensive PCAS care, including coronary angiography, patients with return of spontaneous circulation (ROSC) should be relocated to a different hospital. Even index hospitals that provide PCAS care may not always be able to provide comprehensive post-resuscitation care because of limited resources.

Inter-hospital transfer (IHT) after ROSC is a time-consuming process. When patients who achieve ROSC are transferred to other hospitals, PCAS care can be delayed. The International Liaison Committee on Resuscitation (ILCOR) recommends that emergent coronary angiography (CAG) be performed in OHCA patients with a suspected etiology of cardiac arrest and ST elevation on electrocardiography (ECG) [1]. Several studies have reported good neurological outcomes after transfer to cardiac arrest centers (CACs) [2,3,4]. Therefore, the ILCOR has suggested that all post-arrest patients be transported to a CAC; however, CACs are not always accessible [5,6].

In Korea, patients with sudden cardiac arrest are transported to the closest emergency department (ED) by the emergency medical service (EMS) with basic life support. Because the rate of ROSC before arrival at the ED is low, it is difficult to triage patients on the basis of ECG results after ROSC, and ECG results are usually examined after ROSC at the index hospital. For these reasons, patients who achieve ROSC should be transferred to a CAC for emergent CAG and comprehensive post-resuscitation care. Park et al. reported that the overall rate of IHT was 16.88% among patients who achieved sustained ROSC at an ED in Korea [7]. Previous studies on IHT have focused on the regionalization of cardiac arrest patients. Nevertheless, IHT is needed, and little is known about the neurological outcomes of patients who undergo IHT to a CAC after achieving ROSC. In this study, we sought to determine whether IHT to a CAC after achievement of ROSC was associated with poor outcomes in cardiac arrest patients treated with targeted temperature management (TTM) based on ECG results. We also compared the time from ROSC to TTM induction according to the type of ED visit to determine the effect of delayed TTM on neurological outcomes in post-cardiac arrest patients.

## 2. Methods

### 2.1. Study Design and Population

This was a retrospective analysis of data from a prospective, multicenter, observational cohort study. Data were collected between November 2015 and December 2018 from the Korea Hypothermia Network prospective (KORHN-pro) registry (https://clinicaltrials.gov/:NCT02827422), a multicenter clinical research consortium for TTM in South Korea that was established in 2011. The KORHN-pro manages a web-based prospective registry of OHCA cases treated with TTM to improve post-cardiac-arrest care quality and outcomes. This study and its protocol were approved by the institutional review board of each hospital (IRB No. C2015162(1620)) and were registered on the clinical trial registry platform. Written informed consent was obtained from all enrolled patients. The inclusion criteria were as follows: (1) age ≥ 18 years, (2) OHCA, (3) comatose mental status after ROSC, (4) use of TTM, and (5) ECG performed after achieving ROSC. The exclusion criteria were as follows: (1) hemorrhagic or ischemic stroke confirmed as the cause of cardiac arrest; (2) cerebral performance category (CPC) of 3 or 4 before cardiac arrest; (3) body temperature below 30°C upon arrival; and (4) no provision of PCAS care, including TTM. Enrolled patients received PCAS care according to the protocol specified by each hospital. Many differences exist in the detailed management of PCAS patients among the hospitals, including differences in the devices used, TTM procedures, and shock management procedures; data on relevant variables were collected using a web-based registry. A principal investigator from each participating hospital reviewed the hospital records of OHCA survivors treated with TTM for the following parameters: circulatory status after ROSC, circulatory support, neurological status and neurological examination results, TTM procedure, incidence of complications, and neurological outcomes at discharge and at 1 month and 6 months after ROSC. Three clinical research associates monitored the data and helped to qualify it by sending queries to the investigators. Finally, a data manager examined the data and decided whether the records were acceptable or needed revision.

### 2.2. Variables

All data was prospectively collected from the web-based registry (http://pro.korhn.or.kr) according to the Utstein guidelines [8]. Time variables related to resuscitation included down time (from collapse to ROSC), TTM time (from ROSC to the induction of TTM), and time from ROSC to the achievement of target temperature. We also collected data on variables related to the EMS (witnesses, bystander cardiopulmonary resuscitation, ECG rhythm, and defibrillation) and variables from hospital records (type of ED visit, ECG rhythm, defibrillation, Glasgow coma scale [GCS] score, ECG findings after ROSC, pupillary light reflex [PLR], corneal reflex, mean arterial pressure [MAP], serum lactate levels, CAG within 24 h, and temperature for TTM). After admission to the intensive care unit, sequential organ failure assessment (SOFA) scores were recorded for 7 days.

### 2.3. Statistical Analysis

To assess the differences in hospital visit time and neurological outcomes (good neurological outcome: CPC 1–2 and poor neurological outcome: CPC 3–5), the independent t-test or Mann–Whitney U-test was used for continuous variables and the Pearson’s chi-square or Fisher’s exact test was used for nominal variables. Continuous variables were expressed as the means ± standard deviations, and categorical variables were expressed as numbers and percentages. To compare the differences between patients who directly visited the ED of a CAC and patients transferred from index hospitals, univariate analysis was performed in the same manner. To determine the independent predictive factors for poor neurological outcomes, we performed multivariate logistic regression analysis by initially including variables with a *p*-value < 0.2 and the type of hospital visit and then applying stepwise backward selection to the variables that remained significant. The Hosmer–Lemeshow test was used to evaluate the goodness of fit of the logistic regression model. Adjusted odds ratios (ORs) and 95% confidence intervals (CIs) were generated from the multivariate analyses. In subgroup analysis, patients were assigned to the ST elevation (STE) group or the non-STE group according to the ECG results after ROSC. The TTM time was compared according to the type of hospital visit and neurological outcomes in each subgroup. Significance was set at *p* < 0.05. Statistical analyses were performed using IBM SPSS statistics version 26.0 (IBM Corp., Armonk, NY, USA).

## 3. Results

### 3.1. Baseline Characteristics of the Patients

**Figure 1:**
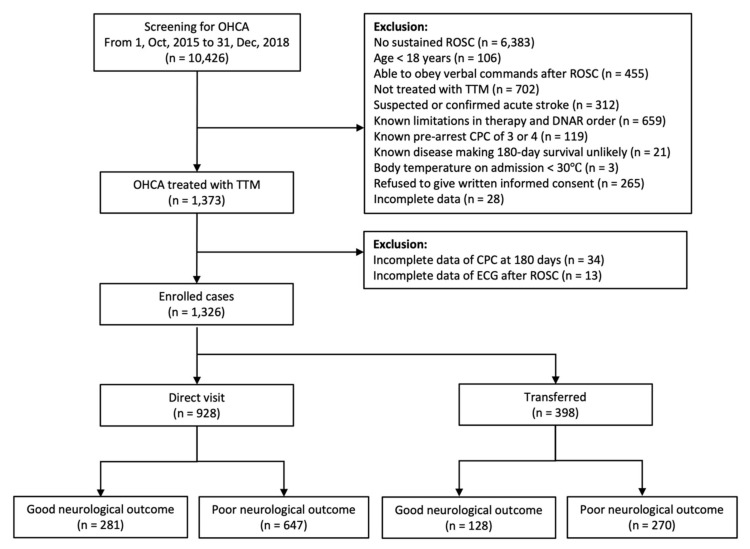
Flow chart describing the enrolment of patients in this study. OHCA: out-of-hospital cardiac arrest; ROSC: return of spontaneous circulation; TTM: targeted temperature management; DNAR: do-not-attempt resuscitation; CPC: cerebral performance category; ECG: electrocardiography.

During the study period, 10426 OHCA patients were screened, and the data of 1373 patients enrolled in 22 hospitals were recorded in the KORHN-pro registry. Of these, 47 patients were excluded because of incomplete data regarding CPC at 6 months or ECG after ROSC. A total of 1326 patients were included in the final study (Figure 1). Of these, 70.0% (928/1326) of patients directly visited the ED and received PCAS care including TTM, and 30.0% (398/1326) of patients were transferred from index hospitals for comprehensive post-resuscitation care.

When variables were compared by the type of hospital visit, no significant differences in neurological outcomes were observed between the direct visit and transfer groups (*p* = 0497; Table 1). Patients in the direct visit group had higher rates of witnessed cardiac arrest and bystander CPR than those in the transfer group (*p* = 0.001). In terms of ECG rhythm assessed by EMS, shockable rhythm was more common in the direct visit group (*p* < 0.001). Down time was not significantly different according to the type of hospital visit (*p* = 0.908). GCS scores, MAP values, and lactate levels after ROSC were higher in the direct visit group than in the transfer group. The time from ROSC to TTM induction and from ROSC to the achievement of the target temperature were longer in the IHT group than in the direct visit group (*p* <0.001). CAG within 24 h after ROSC was performed more often in transferred patients (29.1%) than in direct visit patients (21.6%, *p* = 0.003).

### 3.2. Independent Prognostic Factors for Poor Neurological Outcomes

On univariate analysis for neurological outcomes, there was no statistically significant difference between good and poor neurological outcomes according to the type of hospital visit (*p* = 0.497, Table 2). Down time in the good neurological outcome group was shorter than that in the poor outcome group (*p* < 0.001). There was no significant difference in the time from ROSC to TTM induction (*p* = 0.245), but the time from ROSC to the achievement of the target temperature was longer in the good neurological outcome group than that in the poor neurological outcome group (*p* < 0.001). Analyses of variables including sex, age, pre-arrest CPC, cause of arrest, witness, bystander CPR, ECG rhythm, EMS defibrillation, GCS score after ROSC, PLR, corneal reflex, ECG result after ROSC, CAG within 24 h, lactate level, MAP value, and the highest SOFA score revealed significant differences between the neurological outcome groups.

On multivariate logistic regression analysis for poor neurological outcome, IHT was not statistically significant (OR = 1.044, *p* = 0.536, Table 3). Age, non-cardiac origin of cardiac arrest, non-shockable rhythm, down time, absence of PLX and corneal reflex after ROSC, and the highest SOFA score were found to be independent predictors of poor neurological outcomes.

### 3.3. Time from ROSC to the Induction of TTM According to the Type of Hospital Visit and Neurological Outcomes

ECG after ROSC showed ST elevation in 149 (11.2%) patients. Of these, 126 patients (84.6%) visited the ED and were managed with comprehensive PCAS care, and 26 (17.4%) were transferred from the index hospital after they achieved ROSC. Of 1177 patients showing non-STE results on the ECG after ROSC, 805 (68.4%) visited the ED directly and 372 patients (31.6%) were transferred.

**Figure 2:**
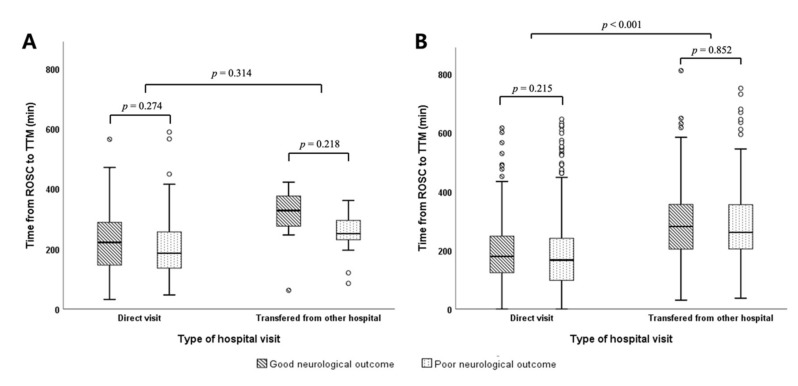
Comparison of the time from return of spontaneous circulation to the induction of targeted temperature management between the direct visit and transfer groups and between the good and poor neurological outcome groups. (**A**) ST elevation group. (**B**) Non-ST elevation group ROSC: return of spontaneous circulation; TTM: targeted temperature management.

In the STE group, there was no significant difference in the time from ROSC to TTM induction between the direct visit and transferred patients (*p* = 0.314, Figure 2A). The TTM time did not differ significantly between those with good and poor neurological outcomes, regardless of hospital visit type (227.73 ± 108.35 vs. 263.63 ± 240.40 min, *p* = 0.274 in the direct visit group; and 312.46 ± 92.51 vs. 261.69 ± 111.29 min, *p* = 0.218 in the transfer group). In the non-STE group, the TTM time among transferred patients was longer than that among direct visit patients (299.22 ± 160.60 min vs 198.77 ± 151.71 min, *p* < 0.001; Table 2). However, there was no significant difference in neurological outcomes according to the TTM time in either the direct visit group (209.39 ± 151.24 min [good outcomes] vs. 194.63 min [poor outcomes], *p* = 0.215) or the transfer group (301.54 ± 151.84 min [good outcomes] vs. 298.18 ± 164.65 min [poor outcomes], *p* = 0.852; Figure 2B).

## 4. Discussion

Treatment of PCAS patients in CACs is beneficial in terms of survival and neurological outcomes. Therefore, it is rational for patients with ROSC at low-volume hospitals to be transferred to CACs for comprehensive PCAS care. Nevertheless, there is a concern that the transfer of patients with ROSC could be harmful because active treatment could be delayed, and adverse events might occur during transport [9,10]. Previous studies on IHT focused on the effect of IHT from low-volume hospitals to high-volume hospitals or from a non-CAC to a CAC [2,3,4,7,11,12,13,14,15]. Therefore, in this study, we investigated the effect of IHT on the neurological outcomes of patients who were treated with comprehensive PCAS care in a CAC. In this study, 30% of patients were transferred from the index hospital (Figure 1). IHT was not an independent predictor of neurological outcomes after 6 months in either the STE or non-STE group (Table 3).

The participating hospitals in this study were generally equipped with the important components of a CAC, such as 24/7 access to interventional cardiology facilities, TTM, diagnostic imaging, and neurologic care. We found that neither IHT itself nor a delay in active PCAS care caused by IHT affected neurological outcomes. In Korea, the public EMS system does not have guidelines regarding the transportation of cardiac arrest patients to a CAC. Usually, the EMS transports OHCA patients to the nearest emergency medical center capable of providing basic and advanced life support. For these reasons, IHT should be required to provide comprehensive post-resuscitation care and to treat the underlying causes of cardiac arrest when cardiac arrest victims achieve ROSC at the index hospital and when the index hospital is a low-volume hospital or cannot provide appropriate treatment. These transfers from low-volume to high-volume hospitals or a non-CAC to a CAC are necessary to improve neurological outcomes. However, few studies have investigated how the delay in comprehensive PCAS care due to IHT could affect the neurotological outcomes of PCAS patients.

Because TTM is a treatment modality for comprehensive PCAS care, we hypothesized that TTM time could serve as an indicator for the implications of comprehensive post-resuscitation care. When patients with ROSC are transferred from index hospitals, physicians consider the possible effects of treatment delay and adverse events during transfer. However, in the present study, we found that IHT after ROSC was not related to poor neurological outcomes at 6 months, even though the TTM time may have been prolonged (Table 1, Figure 2). These findings support the recommendation that cardiac arrest patients with ROSC could be transferred to a CAC as soon as possible when the index hospital cannot provide comprehensive PCAS care and management.

Regionalization of care to specialized centers is important for managing time-critical illnesses by concentrating both services and providers with greater experience [13,15,16,17,18]. Several studies have shown that management after ROSC at high-volume hospitals is associated with better survival; improved neurological outcomes were also seen in patients transferred from low-volume hospitals to high-volume hospitals [2,7]. These findings suggest that the regionalization of care for cardiac arrest results in good neurological outcomes [11,12,19]. On the basis of the results of these studies, the ILCOR recommends transport of all post-arrest patients to a CAC [6]. One of the purposes of regionalization of cardiac arrest care is rapid management of the cause of arrest, especially the use of coronary angiography. Early intervention for coronary artery disease is important for treating PCAS. Several studies have reported on the effects of transfer to a CAC stratified by ECG rhythm (ST elevation or non-ST elevation) [3,4,14,20,21]. In the present study, only 23.8% (316/1326) of patients underwent CAG within 24 h after ROSC (Table 1). Although early CAG within 24 h after ROSC was more common in the STE group (94/149) than in the non-STE group (222/1177), early CAG was not an independent predictor of poor neurological outcomes (Table 3). When regionalization of cardiac arrest care is based on ECG findings after ROSC, confounding may be present due to the interpretation of ST elevation after ROSC. In the present study, only 411 (31%) patients achieved ROSC before arrival at the hospital; 915 (69%) patients had been resuscitated upon arrival to the hospital and achieved ROSC in the ED (Table 1). Regarding these patients, a decision on whether transport them to the nearest emergency medical center or CAC was needed, for early CAG could not be made.

Comprehensive post-resuscitation care is important for improving survival and neurological outcomes in patients achieving ROSC [6]. Regionalization to a CAC could be helpful in providing early comprehensive PCAS care. However, IHT should be required when the index hospital cannot provide PCAS care. We suggest that in these situations, patients who achieve ROSC at the index hospital be transferred to a CAC for comprehensive PCAS care because IHT itself did not show any association with neurological outcomes 6 months after cardiac arrest.

This study has several limitations. The KORHN-pro registry did not include detailed information regarding the index hospitals, such as volume of cardiac arrest patients, the hospitals’ ability to manage PCAS patients, or the reason for transfer after achieving ROSC. Therefore, this was designed as a retrospective study based on the database collected by KORHN prospective observational study. We recorded the TTM time on the basis of ROSC because the cardiac arrest may have occurred while the patient was not under observation, and therefore, the timing of cardiac arrest could have been based on the surmise of the caregiver. In IHT patients, the time for transportation from the index hospital was not recorded in the KORHN-pro registry. Therefore, the transportation time could not be calculated and analyzed for its effect on neurological outcomes. Nevertheless, we compared TTM time (the time from ROSC to TTM induction), including the transportation time from the index hospital to a CAC, to analyze the effect of a delay in active PCAS care in transferred patients.

In conclusion, we found that IHT after achieving ROSC showed no association with neurologic outcomes at 6 months in post-OHCA patients treated with TTM, even though TTM induction was delayed in transferred patients. Therefore, when comprehensive PCAS care is not available at the index hospital, physicians could consider transferring patients with ROSC to a CAC.

## Figures and Tables

**Table 1:** Baseline characteristics of the enrolled patients.

	Direct Visit (n = 928)	Transferred (n = 398)	*p*-Value
Sex			0.996
Male	660 (71.7)	283 (71.1)	
Female	268 (28.9)	115 (28.9)	
Age (year)	58.02 ± 15.81	57.78 ± 15.57	0.797
Pre-arrest CPC			0.678
CPC1	818 (88.1)	354 (88.9)	
CPC2	110 (11.9)	44 (11.1)	
Cause of arrest			0.007
Cardiac origin	597 (64.3)	225 (56.5)	
Non-cardiac origin	331 (35.7)	173 (43.5)	
Witness			0.001
Yes	670 (72.2)	248 (62.3)	
No	251 (27.0)	144 (36.2)	
Unknown	7 (0.8)	6 (1.5)	
Bystander CPR			0.001
Yes	577 (62.2)	239 (60.1)	
No	346 (37.3)	147 (36.9)	
Unknown	5 (0.5)	12 (3.0)	
ECG rhythm in EMS			< 0.001
Shockable	334 (36.0)	110 (27.6)	
Non-shockable	523 (56.4)	194 (48.7)	
Unknown	71 (7.7)	94 (23.6)	
EMS defibrillation			< 0.001
Yes	364 (39.2)	117 (29.4)	
No	411 (44.3)	223 (56.0)	
Unknown	153 (16.5)	58 (14.6)	
Down time (min)	32.59 ± 20.95	32.45 ± 19.19	0.908
ECG rhythm in the ED			< 0.001
Shockable	79 (8.5)	34 (8.5)	
Non-shockable	545 (58.7)	213 (53.5)	
Prehospital ROSC	293 (31.6)	118 (29.6)	
Unknown	11 (1.2)	33 (8.3)	
ED defibrillation			0.81
Yes	166 (17.9)	69 (17.3)	
No	762 (82.1)	329 (82.7)	
GCS score after ROSC			0.035
≤8	901 (97.1)	376 (94.5)	
>8	7 (0.8)	3 (0.8)	
Unknown	20 (2.2)	19 (4.8)	
Pupillary light reflex			0.115
Yes	422 (45.5)	203 (51.0)	
No	499 (53.8)	194 (48.7)	
Unknown	7 (0.8)	1 (0.3)	
Corneal reflex			0.238
Yes	230 (24.8)	85 (21.4)	
No	591 (63.7)	257 (64.6)	
Unknown	107 (11.5)	56 (14.1)	
ECG result after ROSC			< 0.001
ST elevation	123 (13.3)	29 (6.5)	
Non-ST elevation	805 (86.7)	372 (93.5)	
CAG within 24 h			0.003
Yes	200 (21.6)	116 (29.1)	
No	728 (78.4)	282 (70.9)	
Lactate (mmol/L)	10.47 ± 4.94	8.10 ± 5.34	< 0.001
MAP after ROSC (mmHg)	93.61 ± 31.74	88.70 ± 30.41	0.011
Time from ROSC to TTM induction (min)	205.24 ± 158.56	298.42 ± 157.44	< 0.001
Target of TTM (°C)			0.594
≤34	727 (78.3)	317 (79.6)	
>34	201 (21.7)	81 (20.4)	
Time from ROSC to the achievement of the target temperature (min)	423.65 ± 301.33	481.10 ± 245.19	< 0.001
Highest SOFA score in 7 days	12.40 ± 3.68	12.36 ± 3.56	0.863
Neurological outcome after 6 months			0.497
Good (CPC 1-2)	281 (30.3)	128 (32.2)	
Poor (CPC 3-5)	647 (69.7)	270 (67.8)	

Values are expressed as numbers (%) or means ± standard deviations as appropriate. CPC: cerebral performance category; ED: emergency department; CPR: cardiopulmonary resuscitation; EMS: emergency medical service; ECG: electrocardiogram; ROSC: return of spontaneous circulation; GCS: Glasgow coma scale; MAP: mean arterial pressure; TTM: Target temperature management; CAG: coronary angiography; SOFA: sequential organ failure assessment.

**Table 2:** Univariate analysis for neurological outcomes after 6 months.

	Good Neurological Outcomes	Poor Neurological Outcomes	*p*-Value
	(n = 409)	(n = 917)	
Sex			< 0.001
Male	318 (77.8)	625 (68.2)	
Female	91 (22.2)	292 (31.8)	
Age (year)	52.75 ± 14.60	60.26 ± 15.68	< 0.001
Pre-arrest CPC			< 0.001
CPC1	397 (97.1)	775 (84.5)	
CPC2	12 (2.9)	142 (15.5)	
Cause of arrest			< 0.001
Cardiac origin	360 (88.0)	462 (50.4)	
Non-cardiac origin	49 (12.0)	455 (49.6)	
Type of ED visit			0.497
Direct visit	281 (68.7)	647 (70.6)	
Transferred	128 (31.3)	270 (29.4)	
Witness			< 0.001
Yes	343 (83.9)	575 (62.7)	
No	65 (15.9)	330 (36.0)	
Unknown	1 (0.2)	12 (1.3)	
Bystander CPR			0.004
Yes	278 (68.0)	538 (58.7)	
No	125 (30.6)	368 (40.1)	
Unknown	6 (1.5)	11 (1.2)	
ECG rhythm in EMS			< 0.001
Shockable	286 (69.9)	158 (17.2)	
Non-shockable	74 (18.1)	643 (70.1)	
Unknown	49 (12.0)	116 (12.6)	
EMS defibrillation			< 0.001
Yes	284 (69.4)	197 (21.5)	
No	76 (18.6)	558 (60.9)	
Unknown	49 (12.0)	162 (17.7)	
Down time (min)	21.73 ± 16.86	37.37 ± 20.04	< 0.001
ECG rhythm in the ED			< 0.001
Shockable	57 (13.9)	56 (6.1)	
Non-shockable	64 (15.6)	694 (75.7)	
Prehospital ROSC	279 (68.2)	132 (14.4)	
Unknown	9 (2.2)	35 (3.8)	
ED defibrillation			0.482
Yes	77 (18.8)	158 (17.2)	
No	332 (81.2)	759 (82.8)	
GCS score after ROSC			< 0.001
≤8	383 (93.6)	894 (97.5)	
>8	9 (2.2)	1 (0.1)	
Unknown	17 (4.2)	22 (2.4)	
Pupillary light reflex			< 0.001
Yes	334 (81.7)	291 (31.7)	
No	74 (18.1)	619 (67.5)	
Unknown	1 (0.2)	7 (0.8)	
Corneal reflex			< 0.001
Yes	204 (49.9)	111 (12.1)	
No	130 (31.8)	718 (78.3)	
Unknown	75 (18.3)	88 (9.6)	
ECG result after ROSC			< 0.001
ST elevation	159 (38.9)	157 (17.1)	
Non-ST elevation	250 (61.1)	760 (82.9)	
CAG within 24 h			< 0.001
Yes	159 (38.9)	157 (17.1)	
No	250 (61.1)	760 (82.9)	
Lactate (mmol/L)	7.92 ± 5.29	10.56 ± 4.92	< 0.001
MAP after ROSC (mmHg)	100.95 ± 30.84	88.30 ± 30.91	< 0.001
Time from ROSC to TTM induction (min)	241.04 ± 150.45	229.72 ± 169.42	0.245
Target of TTM (°C)			0.882
≤34	321 (78.55)	723 (78.8)	
>34	88 (21.5)	194 (21.2)	
Time from ROSC to the achievement of the target temperature (min)	510.83 ± 290.96	409.47 ± 279.06	< 0.001
Highest SOFA score in 7 days	9.86 ± 3.05	13.52 ± 3.30	< 0.001

Values are expressed as numbers (%) or means ± standard deviations as appropriate. CPC: cerebral performance category; ED: emergency department; CPR: cardiopulmonary resuscitation; EMS: emergency medical service; ECG: electrocardiogram; ROSC: return of spontaneous circulation; GCS: Glasgow coma scale; MAP: mean arterial pressure; TTM: Target temperature management; CAG: coronary angiography; SOFA: sequential organ failure assessment.

**Table 3:** Multivariate regression analysis for poor neurological outcomes after 6 months.

	Odds Ratio	95% CI	*p*-Value
Age (year)	1.044	1.028–1.061	< 0.001
Transferred (vs. direct visit)	0.85	0.508–1.422	0.536
Non-cardiac origin (vs. cardiac origin)	3.297	1.792–6.068	< 0.001
Non-shockable rhythm in EMS (vs. shockable)	6.098	2.569–14.472	< 0.001
Down time (min)	1.042	1.025–1.059	0.001
Non-shockable rhythm in the ED (vs. shockable)	3.082	1.452–6.544	0.003
No pupillary reflex	1.95	1.186–3.206	0.008
No corneal reflex	3.442	2.067–5.733	< 0.001
CAG within 24 h	1.135	0.677–1.903	0.63
MAP after ROSC (mmHg)	0.987	0.980–0.994	< 0.001
Highest SOFA score in 7 days	1.192	1.111–1.278	< 0.001

(CI: confidence interval; EMS: emergency medical service; ED: emergency department; MAP: mean arterial pressure; SOFA: sequential organ failure assessment).
